# 1433. Hand Hygiene Products Consumption and Compliance in Intensive Care Units of 74 Hospitals in Shanghai

**DOI:** 10.1093/ofid/ofad500.1270

**Published:** 2023-11-27

**Authors:** Mengge Han, Hongping Pan, Xiaodong Gao, Bijie Hu

**Affiliations:** Zhongshan University of Fudan University, Shanghai, Shanghai, China; The People's Hospital of Yu xi City, Shanghai, Shanghai, China; Zhongshan Hospital of Fudan University, Shanghai, Shanghai, China; Department of Infectious Diseases, Zhongshan Hospital, Fudan University, Shanghai, Shanghai, China

## Abstract

**Background:**

Understand the hand hygiene products consumption and compliance of the medical personnel in intensive care units (ICUs) of the secondary and tertiary hospitals in Shanghai to provide a scientific basis for hand hygiene monitoring.

**Methods:**

The data was obtained through the Shanghai Hospital Infection Monitoring System from 2017 to 2021. The average daily consumption per bed was caculated for hand hygiene products. The hand hygiene compliance was calculated as the rate of performed disinfections per observed indications in any medical personnel involved in clinical care during an observation period of approximately 20 minutes. Pearson correlation coefficient was used for the correlation analysis of hand hygiene products consumption and compliance rate.

**Results:**

In 105 ICUs of 74 hospitals in Shanghai, 39.85% of medical personnel selected soap and running water, 42.27% used alcohol-based hand rubs, 4.65% used gloves without performing hand hygiene, and 13.22% did not use golves nor perform hand hygiene. The average daily hand hygiene products consumption was 79.24ml per bed , and 46.14 ml and 33.10 ml per bed for soap and alcohol hand rubs, respectively. The hand hygiene products consumption incereasd from 2017 to 2021 (*P*<0.001). The overall hand hygiene compliance rate was 81.64%.The hand hygiene compliance rate was 86.59%, 81.33%, 79.63%, 77.36%, 48.90% and 66.83% for nurses, doctors, continue education personnel/interns, nuring workers, medical technicians and others, respectively, and the rates varied statistically among different personnel (*P*<0.001). The hand hygiene compliance rate incereasd from 2018 to 2021 (*P*<0.001). There was a positive correlation between the average daily hand hygiene products consumption per bed and compliance rate (r=0.703, *P*=0.016).

**Table 1:**
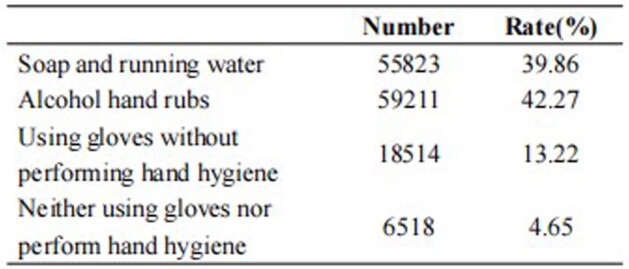
Hand hygiene measures in ICUs of the secondary and tertiary hospitals in Shanghai from 2017-2021

**Figure 1:**
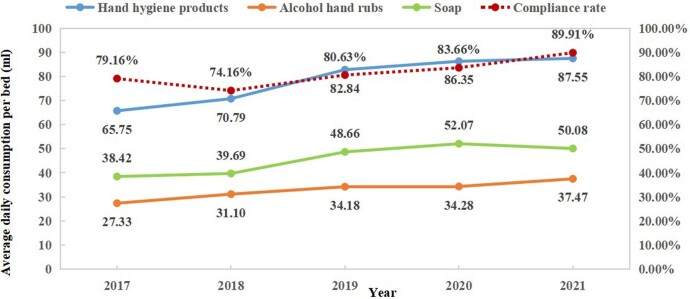
The trends of the hand hygiene products consumption and compliance rates from 2017-2021

**Figure 2:**
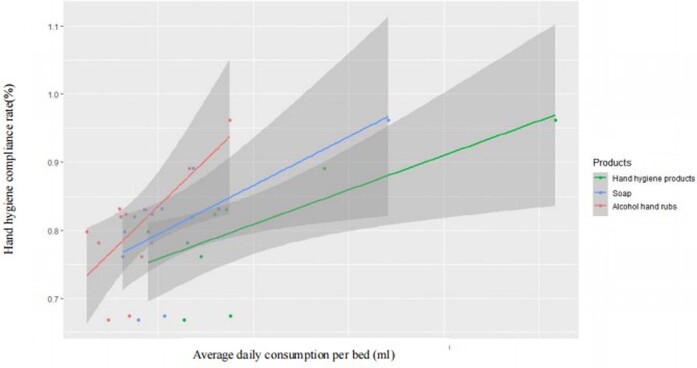
The relationship between hand hygiene products consumption and compliance in ICUs of the secondary and tertiary hospitals in Shanghai from 2017-2021

**Conclusion:**

Both the hand hygiene products consumption and compliance have shown an increasing trend in recent years in Shanghai. And hand hygiene compliance may be evaluated by continuously monitoring hand hygiene products consumption.

**Disclosures:**

**All Authors**: No reported disclosures

